# Supplementary figures and images for: In silico testing of flavonoids as potential inhibitors of protease and helicase domains of dengue and Zika viruses
Source: PeerJ. 2022 Aug 4;10:e13650. doi: 10.7717/peerj.13650 (PMC9357371; doi:10.7717/peerj.13650)

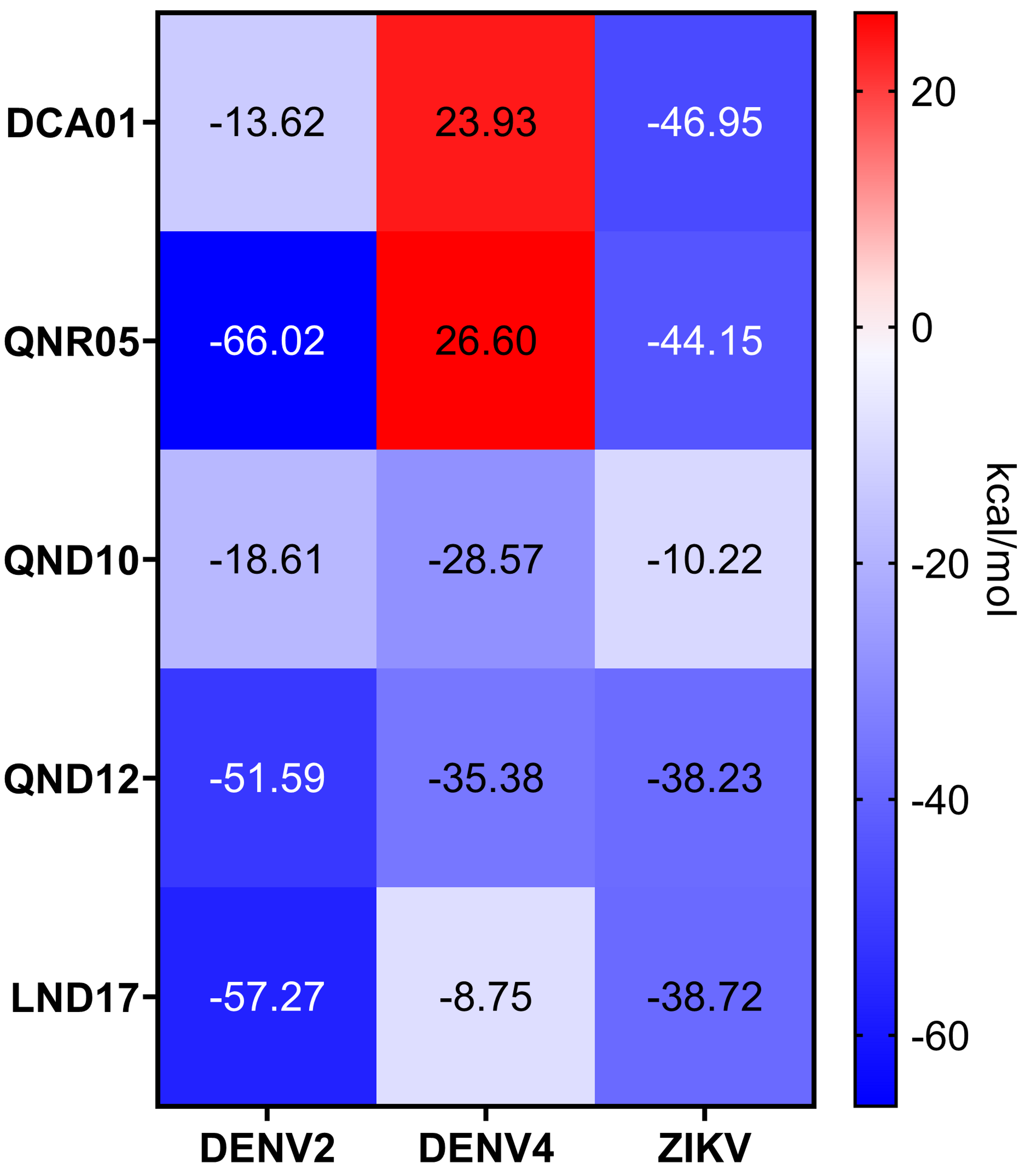

Supplement: Supplemental Information 1 — Calculations were done in UCSF DOCK; plots were obtained using GraphPad Prism v. 8. [file peerj-10-13650-s001.png]

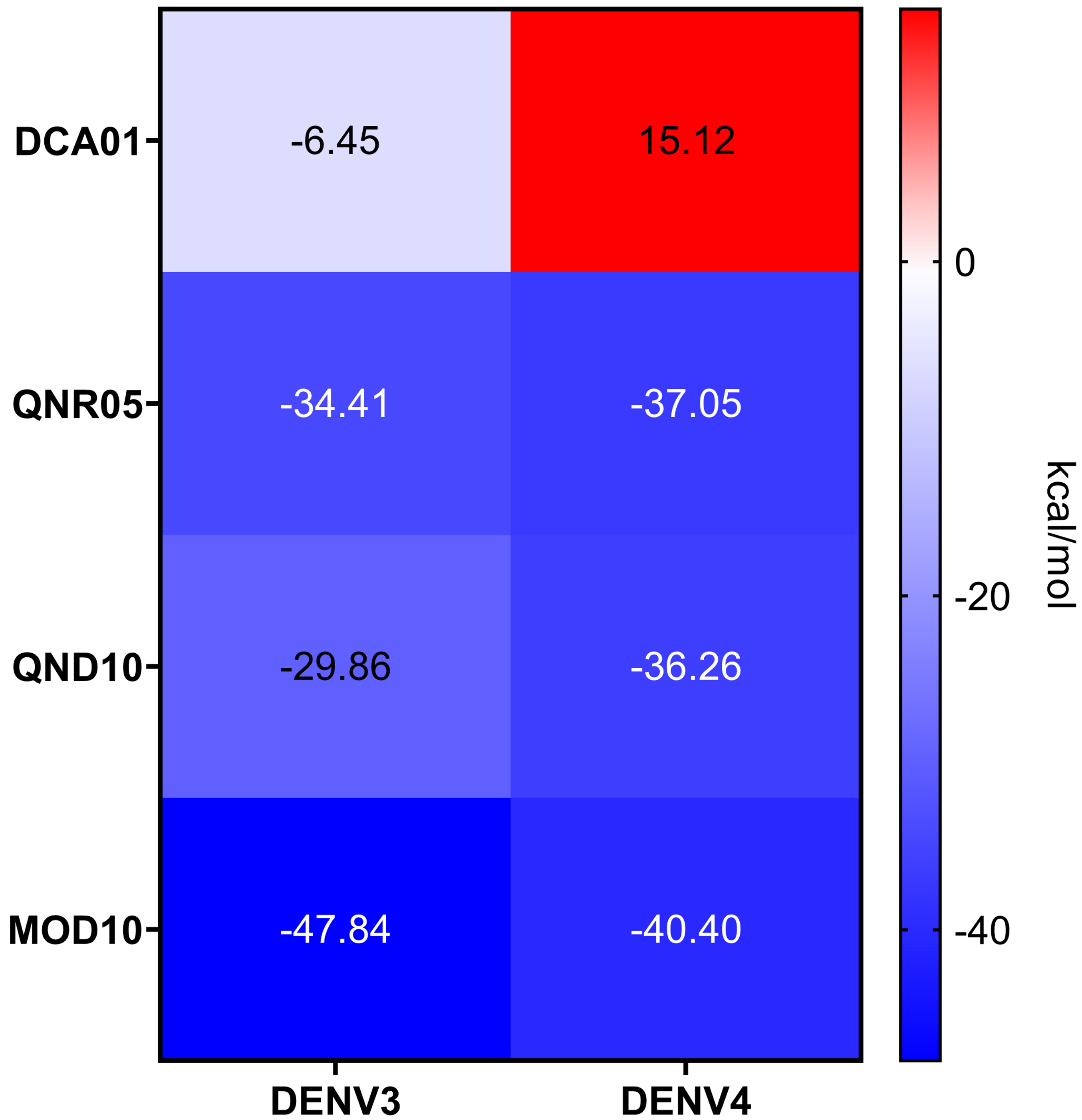

Supplement: Supplemental Information 2 — Calculations were done in UCSF DOCK; plots were obtained using GraphPad Prism v. 8. [file peerj-10-13650-s002.png]

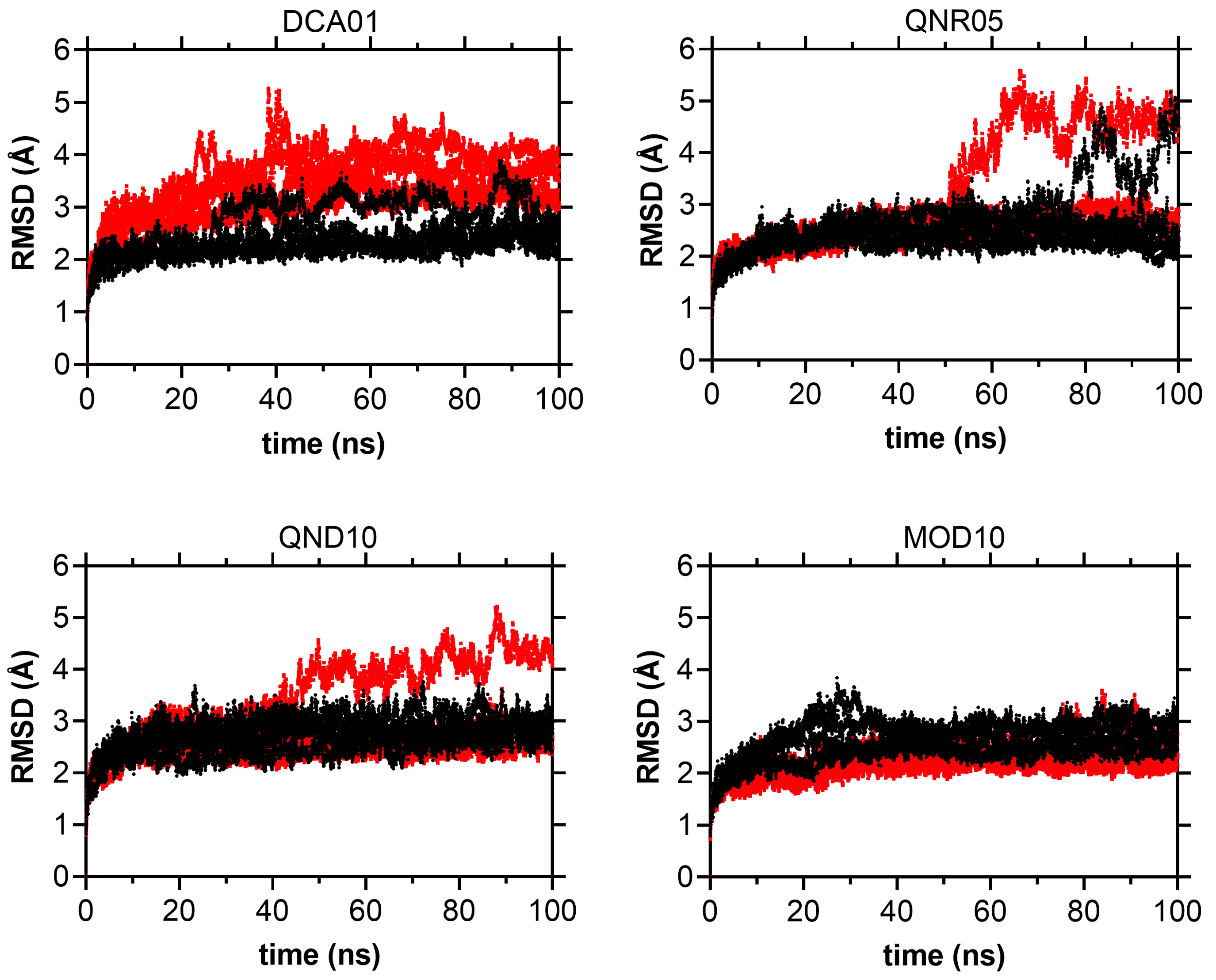

Supplement: Supplemental Information 4 — DENV3 is indicated in black and DENV4 is indicated in red. [file peerj-10-13650-s004.png]

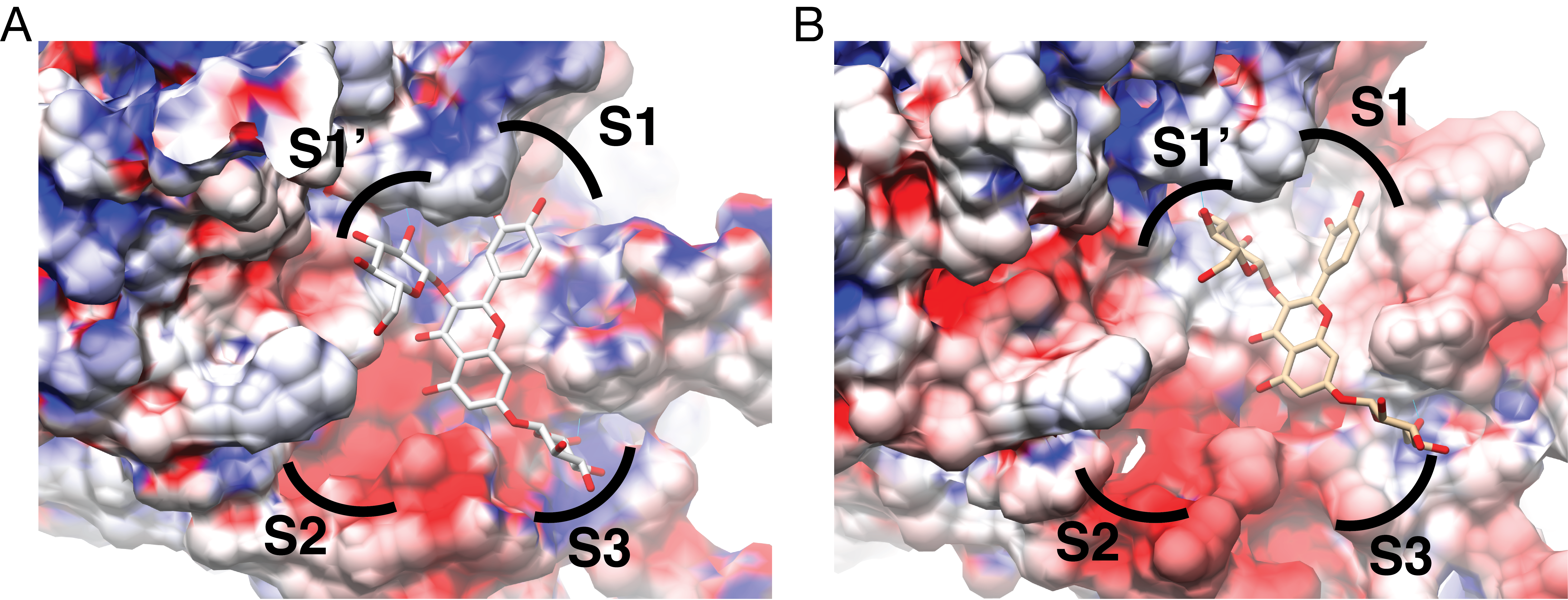

Supplement: Supplemental Information 5 — QND10 bound to (A) DENV3 or (B) DENV4 NS3-pro. Electrostatic scale goes from blue at 5 k bT to red at −5 k bT (Behnam & Klein, 2020) . Electrostatics calculated using APBS, images created in Chimera 1.14 (Pettersen et al., 2004) . [file peerj-10-13650-s005.png]

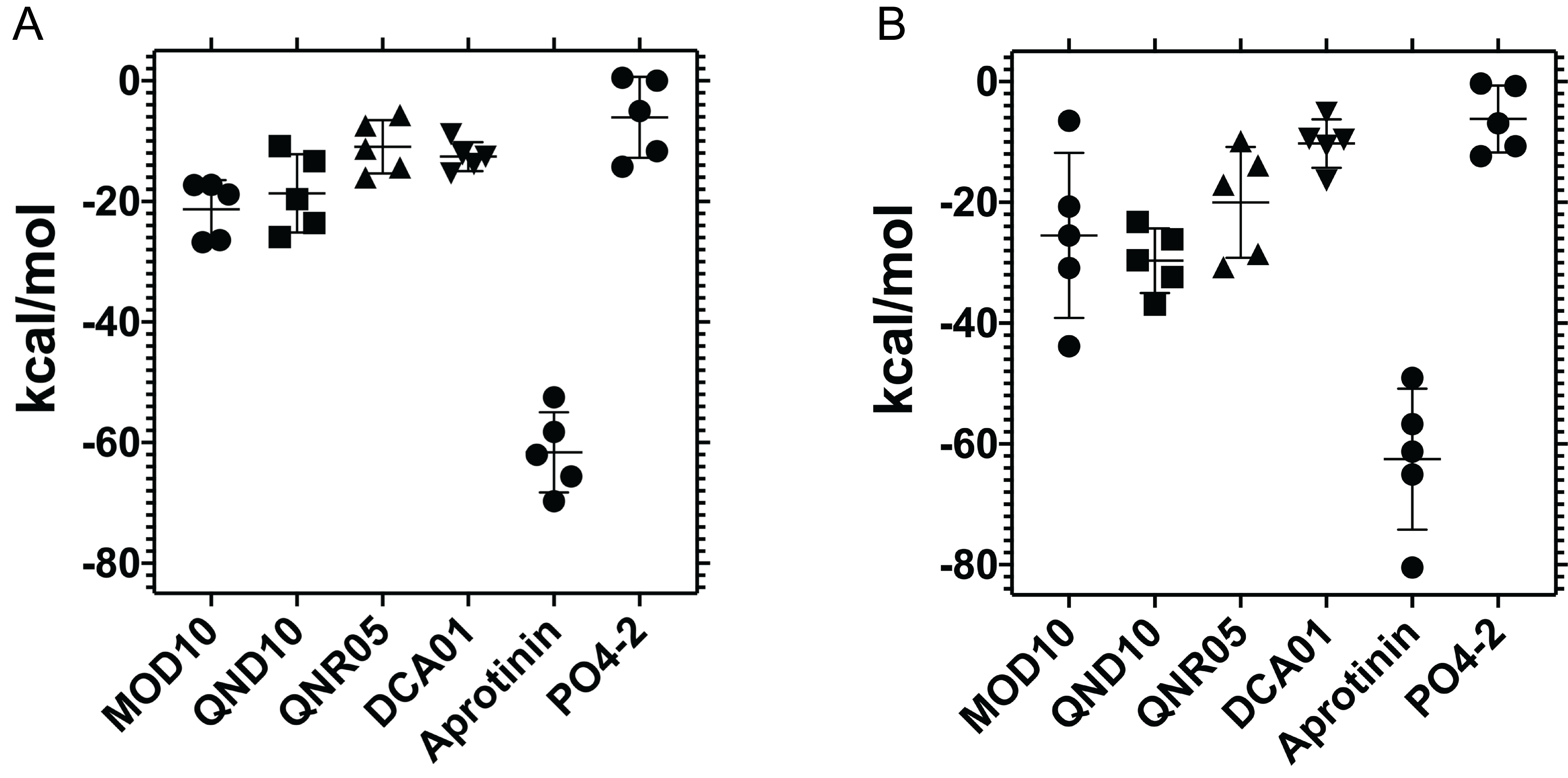

Supplement: Supplemental Information 7 — versus our MOD10, the molecules selected from ADFR docking, the positive aprotinin control and the negative dibasic phosphate control. [file peerj-10-13650-s007.png]
