# Supplementary material for: In silico testing of flavonoids as potential inhibitors of protease and helicase domains of dengue and Zika viruses
Source: PeerJ. 2022 Aug 4;10:e13650. doi: 10.7717/peerj.13650 (PMC9357371; doi:10.7717/peerj.13650)
Supplement: Supplemental Information 8 [file peerj-10-13650-s008.docx]

Table S1. Accession code of the polyprotein sequence for DENV and ZIKV

| _Region_ | _Serotype_ | _ID sequence_ | _Region_ | _Serotype_ | _ID sequence_ | _Region_ | _Serotype_ | _ID sequence_ |
| --- | --- | --- | --- | --- | --- | --- | --- | --- |
| **_Africa_** | _DENV1_ | _AGW21594_ | **_Asia_** | _DENV1_ | _BBG31501_ | **_Europa_** | _DENV1_ | _CCG93466_ |
|  |  | _AXS75992_ |  |  | _AUW27526_ |  |  | _AIN75463_ |
|  |  | _ANC57584_ |  |  | _AXX75607_ |  |  | _AVY51407_ |
|  |  | _AAK60418_ |  |  | _AXX75606_ |  |  | _-_ |
|  |  | _ABC07332_ |  |  | _AUW27528_ |  |  | _-_ |
|  |  | _ABC07331_ |  |  | _AUW27527_ |  |  | _-_ |
|  | _DENV2_ | _AVH78234_ |  | _DENV2_ | _AXU25276_ |  | _DENV2_ | _AVY51408_ |
|  |  | _AVH78235_ |  |  | _AXU25284_ |  |  | _AED98520_ |
|  |  | _AVH78236_ |  |  | _BBG31505_ |  |  | _-_ |
|  |  | _AVH78237_ |  |  | _BBG31506_ |  |  | _-_ |
|  |  | _AVH78239_ |  |  | _BBG31509_ |  |  | _-_ |
|  |  | _AVQ12083_ |  |  | _AZS59400_ |  |  | _-_ |
|  | _DENV3_ | _ACQ44384_ |  | _DENV3_ | _BBG31513_ |  | _DENV3_ | _-_ |
|  |  | _ANC57608_ |  |  | _AXX75610_ |  |  | _-_ |
|  |  | _-_ |  |  | _AXX75611_ |  |  | _-_ |
|  |  | _-_ |  |  | _ASK05287_ |  |  | _-_ |
|  |  | _-_ |  |  | _ASX95468_ |  |  | _-_ |
|  |  | _-_ |  |  | _BBG31511_ |  |  | _-_ |
|  | _DENV4_ | _AVY51410_ |  | _DENV4_ | _BBG31520_ |  | _DENV4_ | _-_ |
|  |  | _AHK05342_ |  |  | _BBG31521_ |  |  | _-_ |
|  |  | _-_ |  |  | _BBG31514_ |  |  | _-_ |
|  |  | _-_ |  |  | _BBG31515_ |  |  | _-_ |
|  |  | _-_ |  |  | _BBG31516_ |  |  | _-_ |
|  |  | _-_ |  |  | _BBG31517_ |  |  | _-_ |
|  | _ZIKV_ | _QDH45910_ |  | _ZIKV_ | _BBC70847_ |  | _ZIKV_ | _AMS00611_ |
|  |  | _AEN75265_ |  |  | _AZS35407_ |  |  | _AMN14619_ |
|  |  | _AMR68906_ |  |  | _QGA72951_ |  |  | _ART29823_ |
|  |  | _AZS35403_ |  |  | _QDZ58858_ |  |  | _ART29825_ |
|  |  | _AZS35404_ |  |  | _AVG19203_ |  |  | _ART29828_ |
|  |  | _AZS35405_ |  |  | _APH11587_ |  |  | _AOX49265_ |
|  |  |  |  |  |  |  |  |  |
| **_N. America_** | _DENV1_ | _ACW82969_ | **_Oceania_** | _DENV1_ | _AVM80382_ | **_S. America_** | _DENV1_ | _AXB26588_ |
|  |  | _ADA60793_ |  |  | _AYP31256_ |  |  | _AXB26592_ |
|  |  | _ALJ53459_ |  |  | _AYP31257_ |  |  | _AXB26594_ |
|  |  | _AHI43751_ |  |  | _AXS75990_ |  |  | _AKQ00017_ |
|  |  | _AHC98425_ |  |  | _AMN88557_ |  |  | _AUN35113_ |
|  |  | _AHC98426_ |  |  | _AFY10029_ |  |  | _AUN35112_ |
|  | _DENV2_ | _AOE23002_ |  | _DENV2_ | _AYO90660_ |  | _DENV2_ | _AWI47685_ |
|  |  | _APW84878_ |  |  | _AYO90661_ |  |  | _AWK29893_ |
|  |  | _AHI43964_ |  |  | _ANY58850_ |  |  | _AWK29892_ |
|  |  | _AHI43753_ |  |  | _ANY58849_ |  |  | _AUN35129_ |
|  |  | _ANC57593_ |  |  | _ACQ44517_ |  |  | _AUN35128_ |
|  |  | _AOE23003_ |  |  | _ANT47238_ |  |  | _AUN35135_ |
|  | _DENV3_ | _AHC98449_ |  | _DENV3_ | _AVD96674_ |  | _DENV3_ | _AXG22240_ |
|  |  | _AHC98450_ |  |  | _AVD96676_ |  |  | _AXB26587_ |
|  |  | _AHC98451_ |  |  | _AVD96677_ |  |  | _AXB26589_ |
|  |  | _AHC98457_ |  |  | _AVD96678_ |  |  | _AXB26590_ |
|  |  | _AHC98458_ |  |  | _AFN80339_ |  |  | _AXB26602_ |
|  |  | _ACQ44481_ |  |  | _AVD96675_ |  |  | _ACO06174_ |
|  | _DENV4_ | _ALJ53458_ |  | _DENV4_ | _AXG50965_ |  | _DENV4_ | _AUN35144_ |
|  |  | _AMW17747_ |  |  | _AFY10033_ |  |  | _AKQ00034_ |
|  |  | _ACQ44406_ |  |  | _AFY10039_ |  |  | _AKQ00035_ |
|  |  | _ACQ44407_ |  |  | _AFY10037_ |  |  | _AKQ00036_ |
|  |  | _ACQ44408_ |  |  | _AFY10035_ |  |  | _AKQ00037_ |
|  |  | _ACH61714_ |  |  | _AFY10036_ |  |  | _AXB26600_ |
|  | _ZIKV_ | _AMQ34003_ |  | _ZIKV_ | _AOI20067_ |  | _ZIKV_ | _ARU07076_ |
|  |  | _AMQ34004_ |  |  | _ANO46309_ |  |  | _ARU07075_ |
|  |  | _AQW34707_ |  |  | _ANO46307_ |  |  | _AML81028_ |
|  |  | _APB03021_ |  |  | _ANO46303_ |  |  | _AZS35360_ |
|  |  | _AOS90221_ |  |  | _AVG19275_ |  |  | _AZS35374_ |
|  |  | _ASL68979_ |  |  | _ARB08102_ |  |  | _APY24200_ |
