# Supplementary material for: In silico testing of flavonoids as potential inhibitors of protease and helicase domains of dengue and Zika viruses
Source: PeerJ. 2022 Aug 4;10:e13650. doi: 10.7717/peerj.13650 (PMC9357371; doi:10.7717/peerj.13650)
Supplement: Supplemental Information 9 [file peerj-10-13650-s009.docx]

Table S2. Accession code of the crystal structure of NS3 protein for DENV and ZIKV

| Virus | Code | Domain | |
| --- | --- | --- | --- |
|  |  | Protease | Helicase |
| DENV1 | [3L6P](https://www.rcsb.org/structure/3L6P) | 2.3 Å | - |
| DENV2 | [2FOM](https://www.rcsb.org/structure/2FOM) | 2.2 Å | - |
| DENV3 | [3U1I](https://www.rcsb.org/structure/3U1I) | 1.5 Å | - |
| DENV4 | [5YVV](https://www.rcsb.org/structure/5YVV) | 3.1 Å | - |
| ZIKV | [5YOD](https://www.rcsb.org/structure/5YOD) | 1.9 Å | - |
| DENV2 | [2BMF](https://www.rcsb.org/structure/2BMF) | - | 2.4 Å |
| DENV4 | [2JLS](https://www.rcsb.org/structure/2JLS) | - | 2.2 Å |
| ZIKV | [5TXG](https://www.rcsb.org/structure/5TXG) | - | 2.0 Å |
