# Supplementary material for: In silico testing of flavonoids as potential inhibitors of protease and helicase domains of dengue and Zika viruses
Source: PeerJ. 2022 Aug 4;10:e13650. doi: 10.7717/peerj.13650 (PMC9357371; doi:10.7717/peerj.13650)
Supplement: Supplemental Information 10 [file peerj-10-13650-s010.docx]

Table S3. Polyprotein residue sequence identity, in percentage, for DENV and ZIKV (3430 aa aligned).

|  | DENV1 | DENV2 | DENV3 | DENV4 | ZIKV |
| --- | --- | --- | --- | --- | --- |
| DENV1 | 96.29-100.0 |  |  |  |  |
| DENV2 | 71.54-72.56 | 85.52-100.0 |  |  |  |
| DENV3 | 77.60-78.42 | 71.70-72.76 | 97.28-100.0 |  |  |
| DENV4 | 68.14-69.31 | 68.83-70.10 | 69.54-70.31 | 96.25-100.0 |  |
| ZIKV | 54.62-55.38 | 53.88-55.54 | 55.65-56.24 | 55.35-56.05 | 95.91-100.0 |
