# Supplementary material for: In silico testing of flavonoids as potential inhibitors of protease and helicase domains of dengue and Zika viruses
Source: PeerJ. 2022 Aug 4;10:e13650. doi: 10.7717/peerj.13650 (PMC9357371; doi:10.7717/peerj.13650)
Supplement: Supplemental Information 11 [file peerj-10-13650-s011.docx]

Table S4. NS3 residue sequence identity, in percentage, for DENV and ZIKV (618 aa aligned)

|  | DENV1 | DENV2 | DENV3 | DENV4 | ZIKV |
| --- | --- | --- | --- | --- | --- |
| DENV1 | 96.28-100 |  |  |  |  |
| DENV2 | 78.51-80.93 | 90.77-100 |  |  |  |
| DENV3 | 84.49-86.10 | 79.81-81.90 | 97.90-100 |  |  |
| DENV4 | 75.92-77.06 | 76.69-79.61 | 78.35-79.64 | 97.57-100.0 |  |
| ZIKV | 64.94-66.55 | 65.10-66.88 | 66.39-67.52 | 66.39-68.01 | 96.76-100 |
