# Supplementary material for: In silico testing of flavonoids as potential inhibitors of protease and helicase domains of dengue and Zika viruses
Source: PeerJ. 2022 Aug 4;10:e13650. doi: 10.7717/peerj.13650 (PMC9357371; doi:10.7717/peerj.13650)
Supplement: Supplemental Information 12 [file peerj-10-13650-s012.docx]

Tables S5. NS3-hel domain residue sequence identity, in percentage, for DENV and ZIKV (438 aa aligned).

|  | DENV1 | DENV2 | DENV3 | DENV4 | ZIKV |
| --- | --- | --- | --- | --- | --- |
| DENV1 | 97.05-100 |  |  |  |  |
| DENV2 | 82.19-84.28 | 92.71-100 |  |  |  |
| DENV3 | 88.86-90.68 | 84.51-86.56 | 97.95-100 |  |  |
| DENV4 | 80.82-82.00 | 81.51-84.05 | 82.46-84.05 | 98.63-100 |  |
| ZIKV | 69.63-70.78 | 69.57-72.31 | 70.78-72.15 | 70.71-72.02 | 97.27-100 |
