# Supplementary material for: In silico testing of flavonoids as potential inhibitors of protease and helicase domains of dengue and Zika viruses
Source: PeerJ. 2022 Aug 4;10:e13650. doi: 10.7717/peerj.13650 (PMC9357371; doi:10.7717/peerj.13650)
Supplement: Supplemental Information 13 [file peerj-10-13650-s013.docx]

Table S6. NS3-pro domain residue sequence identity, in percentage, for DENV and ZIKV (180 aa aligned).

|  | DENV1 | DENV2 | DENV3 | DENV4 | ZIKV |
| --- | --- | --- | --- | --- | --- |
| DENV1 | 93.29-100 |  |  |  |  |
| DENV2 | 66.48-73.48 | 84.91-100 |  |  |  |
| DENV3 | 73.18-76.53 | 68.71-72.62 | 97.20-100 |  |  |
| DENV4 | 62.57-67.03 | 64.24-68.71 | 67.03-69.27 | 94.41-100 |  |
| ZIKV | 51.39-56.42 | 51.95-55.30 | 53.07-57.54 | 54.74-58.65 | 94.41-100 |
