# Supplementary material for: In silico testing of flavonoids as potential inhibitors of protease and helicase domains of dengue and Zika viruses
Source: PeerJ. 2022 Aug 4;10:e13650. doi: 10.7717/peerj.13650 (PMC9357371; doi:10.7717/peerj.13650)
Supplement: Supplemental Information 14 [file peerj-10-13650-s014.docx]

Table S7. Analysis of residue conservation in: alpha helix 1’ (residues 365-379), alpha helix 2’ (residues 390-400) of domain II and alpha helix 7’’ (residues 525-537) and alpha helix 3’’ (residues 602-615) of domain III, as part of the RNA-binding zone for DENV and ZIKV. All values are percentages.

| α1’ DII (Consensus sequence: SIKAGNDIANCLRKN) | | | | | |
| --- | --- | --- | --- | --- | --- |
|  | DENV1 | DENV2 | DENV3 | DENV4 | ZIKV |
| DENV1 | 93.33-100 |  |  |  |  |
| DENV2 | 80-93.33 | 80-100 |  |  |  |
| DENV3 | 86.66-100 | 80-93.33 | 93.33-100 |  |  |
| DENV4 | 86.66-93.33 | 80-86.66 | 86.66-93.33 | 100 |  |
| ZIKV | 53.33 | 60-66.66 | 53.33 | 53.33 | 100 |
|  | | | | | |
| α2’ DII (Consensus sequence: TFDTEYQKTKL) | | | | | |
|  | DENV1 | DENV2 | DENV3 | DENV4 | ZIKV |
| DENV1 | 100.00 |  |  |  |  |
| DENV2 | 63.63-81.81 | 72.72-100 |  |  |  |
| DENV3 | 81.81-90.90 | 54.54-72.72 | 81.81-100 |  |  |
| DENV4 | 81.81 | 63.63-72.72 | 81.81-90.90 | 100 |  |
| ZIKV | 72.72-81.81 | 45.45-63.63 | 63.63-81.81 | 63.63 | 90.90-100 |
|  |  |  |  |  |  |
| α7” DIIΙ (Consensus sequence: RLRGEARKTFVEL) | | | | | |
|  | DENV1 | DENV2 | DENV3 | DENV4 | ZIKV |
| DENV1 | 100 |  |  |  |  |
| DENV2 | 92.30 | 84.61-100 |  |  |  |
| DENV3 | 84.61 | 76.92-92.30 | 100 |  |  |
| DENV4 | 92.30 | 84.61-92.30 | 84.61 | 100 |  |
| ZIKV | 76.92 | 69.23-76.92 | 69.23 | 84.61 | 100 |
|  |  |  |  |  |  |
| α3” DIIΙ (Consensus sequence: YSDPLALKEFKEFAA) | | | | | |
|  | DENV1 | DENV2 | DENV3 | DENV4 | ZIKV |
| DENV1 | 93.33-100 |  |  |  |  |
| DENV2 | 73.33-93.33 | 86.66-100 |  |  |  |
| DENV3 | 73.33-93.33 | 73.33-100 | 86.66-100 |  |  |
| DENV4 | 60-66.66 | 73.33-80 | 66.66-73.33 | 100 |  |
| ZIKV | 60-66.66 | 73.33 | 60-73.33 | 60 | 100 |
