# Supplementary material for: In silico testing of flavonoids as potential inhibitors of protease and helicase domains of dengue and Zika viruses
Source: PeerJ. 2022 Aug 4;10:e13650. doi: 10.7717/peerj.13650 (PMC9357371; doi:10.7717/peerj.13650)
Supplement: Supplemental Information 15 [file peerj-10-13650-s015.docx]

Table S8. Structural similarity matrix, in Å, of the RNA-binding site of three structures of the helicase domain (alpha helices of domain II and III).

|  | DENV2 | DENV4 | ZIKV |
| --- | --- | --- | --- |
| DENV2 | 0 |  |  |
| DENV4 | 0.831 | 0 |  |
| ZIKV | 1.144 | 1.254 | 0 |
