# Supplementary material for: In silico testing of flavonoids as potential inhibitors of protease and helicase domains of dengue and Zika viruses
Source: PeerJ. 2022 Aug 4;10:e13650. doi: 10.7717/peerj.13650 (PMC9357371; doi:10.7717/peerj.13650)
Supplement: Supplemental Information 16 [file peerj-10-13650-s016.docx]

Table S9. Percent identity matrix of the catalytic triad H51, D75 and S135 plus 5 adjacent residues for DENV and ZIKV

| H51 and 5 adjacent residues (consensus sequence: FHTMWHVTRGA) | | | | | |
| --- | --- | --- | --- | --- | --- |
|  | DENV1 | DENV2 | DENV3 | DENV4 | ZIKV |
| DENV1 | 90.90-100 |  |  |  |  |
| DENV2 | 90.90-100 | 90.90-100 |  |  |  |
| DENV3 | 90.90-100 | 90.90-100 | 100 |  |  |
| DENV4 | 90.90-100 | 90.90-100 | 90.90 | 100 |  |
| ZIKV | 81.81-90.91 | 81.81-100 | 81.81-90.90 | 81.81-90.90 | 90.90-100 |
|  |  |  |  |  |  |
| D75 and 5 adjacent residues (Consensus sequence: ADVKKDLISYG) | | | | | |
|  | DENV1 | DENV2 | DENV3 | DENV4 | ZIKV |
| DENV1 | 90.90-100 |  |  |  |  |
| DENV2 | 72.72-90.90 | 90.90-100 |  |  |  |
| DENV3 | 90.90-100 | 81.81-90.90 | 100 |  |  |
| DENV4 | 54.54-63.63 | 72.72-81.81 | 63.63 | 90.90-100 |  |
| ZIKV | 54.54-63.63 | 54.54-63.63 | 54.54 | 45.45 | 100 |
|  |  |  |  |  |  |
| H135 and 5 adjacent residues (Consensus sequence: FKPGTSGSPIV) | | | | | |
|  | DENV1 | DENV2 | DENV3 | DENV4 | ZIKV |
| DENV1 | 90.90-100 |  |  |  |  |
| DENV2 | 81.81-90.90 | 81.81-100 |  |  |  |
| DENV3 | 90.90 | 81.81-90.90 | 100 |  |  |
| DENV4 | 90.90-100 | 81.81-90.90 | 90.90-100 | 90.90-100 |  |
| ZIKV | 54.55-72.72 | 54.54-63.63 | 54.55-63.64 | 54.54-63.63 | 90.90-100 |
