# Supplementary material for: In silico testing of flavonoids as potential inhibitors of protease and helicase domains of dengue and Zika viruses
Source: PeerJ. 2022 Aug 4;10:e13650. doi: 10.7717/peerj.13650 (PMC9357371; doi:10.7717/peerj.13650)
Supplement: Supplemental Information 17 [file peerj-10-13650-s017.docx]

Table S10. Structural similarity matrix, in Å. of the active site of five protease domain structures (catalytic triad and 5 adjacent amino acids).

|  | DENV1 | DENV2 | DENV3 | DENV4 | ZIKV |
| --- | --- | --- | --- | --- | --- |
| DENV1 | 0 |  |  |  |  |
| DENV2 | 0.638 | 0 |  |  |  |
| DENV3 | 0.43 | 0.645 | 0 |  |  |
| DENV4 | 0.79 | 0.833 | 0.699 | 0 |  |
| ZIKV | 0.6737 | 0.632 | 0.513 | 0.744 | 0 |
