# Supplementary material for: In silico testing of flavonoids as potential inhibitors of protease and helicase domains of dengue and Zika viruses
Source: PeerJ. 2022 Aug 4;10:e13650. doi: 10.7717/peerj.13650 (PMC9357371; doi:10.7717/peerj.13650)
Supplement: Supplemental Information 19 — It contains the access codes of each of the 20 ligands used in the study, through a hyperlink that redirects to the PubChem database, showing the individual characteristics of each molecule. [file peerj-10-13650-s019.docx]

**Supplementary information for**

***In silico* testing of flavonoids as potential inhibitors of protease and helicase domains of dengue and Zika viruses**

Omar Cruz-Arreola^1^, Abdú Orduña-Díaz^2^, Fabiola Domínguez^3^, Julio Reyes-Leyva^1^, Verónica Vallejo-Ruiz^1^, Lenin Domínguez-Ramírez^4^*, Gerardo Santos-López^1^*.

*^1^Laboratorio de Biología Molecular y Virología, Centro de Investigación Biomédica de Oriente, Instituto Mexicano del Seguro Social, Metepec, Atlixco, Puebla, México.*

*^2^Centro de Investigación en Biotecnología Aplicada (CIBA), Instituto Politécnico Nacional, Tepetitla, Tlaxcala, México.*

*^3^Laboratorio de Biotecnología de Productos Naturales, Centro de Investigación Biomédica de Oriente, Instituto Mexicano del Seguro Social, Metepec, Atlixco, Puebla, México.*

*^4^Department of Chemical and Biological Sciences, School of Sciences, Universidad de las Américas Puebla, San Andrés Cholula, Puebla, Mexico.*

*Laboratorio de Biología Molecular y Virología, Centro de Investigación Biomédica de Oriente, Instituto Mexicano del Seguro Social, Metepec, Atlixco, Puebla, México.*

***Corresponding Author**

Dr. Lenin Domínguez-Ramírez

[julio.dominguez@udlap.mx](mailto:julio.dominguez@udlap.mx)

Gerardo Santos-López

gerardo.santos.lopez@gmail.com

Compounds Selected for Molecular docking Assays for NS3-hel and NS3-pro

| Molecule Code | Molecule name | Substance SID | Chemical structure |
| --- | --- | --- | --- |
| **DCA01** | [3,5-Dicaffeoylquinic Acid](https://pubchem.ncbi.nlm.nih.gov/compound/6474310) | 6474310 | 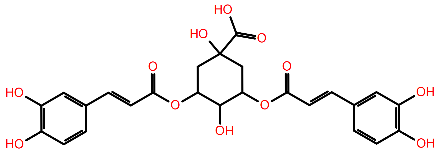 |
| **CAA02** | [Caffeic Acid](https://pubchem.ncbi.nlm.nih.gov/compound/689043) | 689043 | 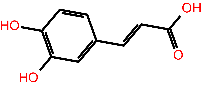 |
| **CFA03** | [5-O-Caffeoylquinic Acid](https://pubchem.ncbi.nlm.nih.gov/compound/5280633) | 5280633 | 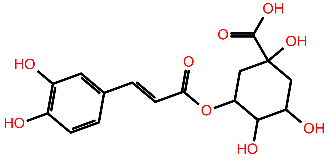 |
| **LNG04** | [Luteolin-7-O-Glucoside](https://pubchem.ncbi.nlm.nih.gov/compound/5280637) | 5280637 | 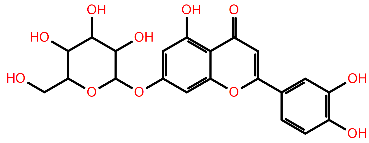 |
| **QNR05** | [Quercetin 3-Rutinoside](https://pubchem.ncbi.nlm.nih.gov/compound/5280805) | 5280805 | 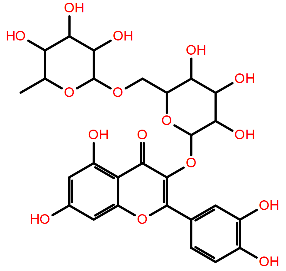 |
| **CHA06** | [Chicoric Acid](https://pubchem.ncbi.nlm.nih.gov/compound/5281764) | 5281764 | 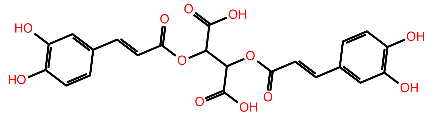 |
| **QND07** | [Quercetin 3,4'-O-Diglucoside](https://pubchem.ncbi.nlm.nih.gov/compound/5320835) | 5320835 | 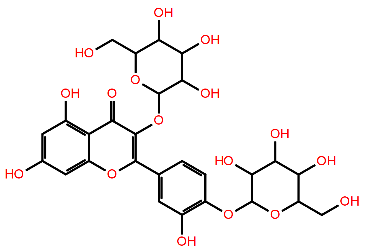 |
| **KFN08** | [Kaempferitrin](https://pubchem.ncbi.nlm.nih.gov/compound/5486199) | 5486199 | 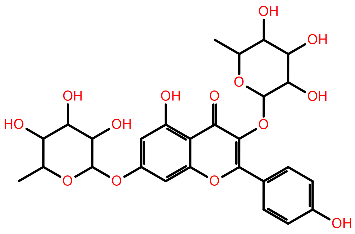 |
| **DCA09** | [4,5-Di-O-Caffeoylquinic Acid](https://pubchem.ncbi.nlm.nih.gov/compound/6474309) | 6474309 | 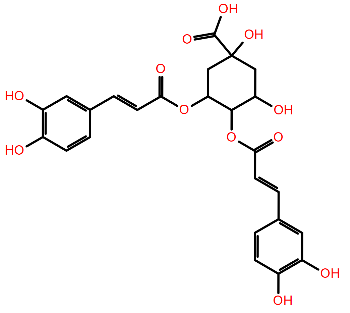 |
| **QND10** | [Quercetin 3,7-Diglucoside](https://pubchem.ncbi.nlm.nih.gov/compound/10121947) | 10121947 | 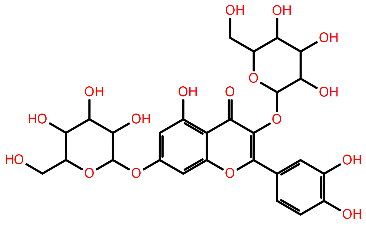 |
| **QND11** | [Quercetin 3-Diglucoside](https://pubchem.ncbi.nlm.nih.gov/compound/10211337) | 10211337 | 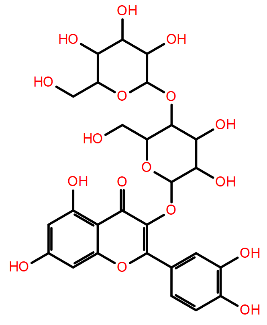 |
| **QND12** | [Quercetin 7,4'-Diglucoside](https://pubchem.ncbi.nlm.nih.gov/compound/11968881) | 11968881 | 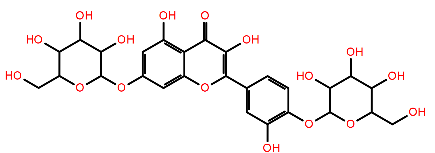 |
| **LNR13** | [Luteolin-7-O-Rutinoside](https://pubchem.ncbi.nlm.nih.gov/compound/14032966) | 14032966 | 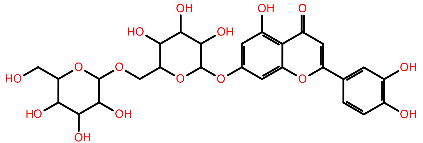 |
| **QND14** | [Quercetin 3,5-O-Diglucoside](https://pubchem.ncbi.nlm.nih.gov/compound/44229098) | 44229098 | 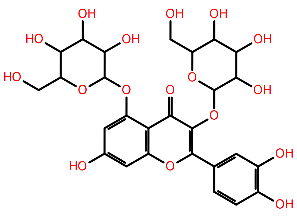 |
| **LND15** | [Luteolin 7,3'-Diglucoside](https://pubchem.ncbi.nlm.nih.gov/compound/44258089) | 44258089 | 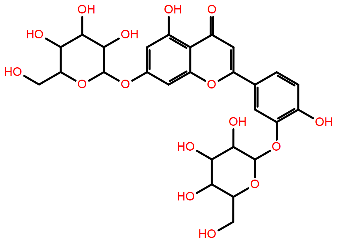 |
| **LND16** | [Luteolin 7,4'-Diglucoside](https://pubchem.ncbi.nlm.nih.gov/compound/44258093) | 44258093 | 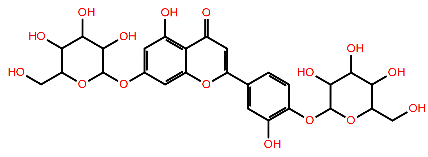 |
| **LND17** | [Luteolin 3',4'-Diglucosid](https://pubchem.ncbi.nlm.nih.gov/compound/44258099) | 44258099 | 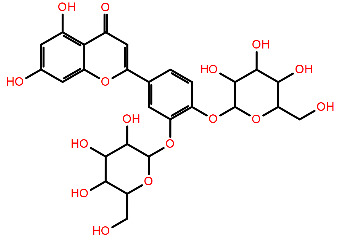 |
| **QND18** | [Quercetin 3,3'-Diglucoside](https://pubchem.ncbi.nlm.nih.gov/compound/44259153) | 44259153 | 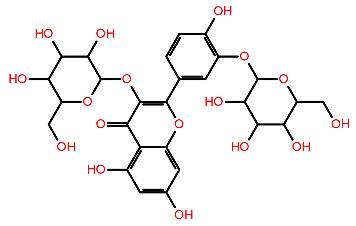 |
| **KFG19** | [Kaempferol 3-Glucoside](https://pubchem.ncbi.nlm.nih.gov/compound/5282102) | 5282102 | 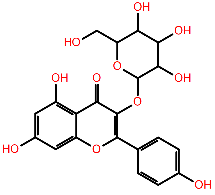 |
| **QNG20** | [Quercetin 3-Galactoside](https://pubchem.ncbi.nlm.nih.gov/compound/5281643) | 5281643 | 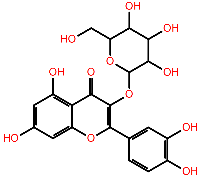 |
